# False positive results of Bowie and Dick type test used for hospital steam sterilizer with slower come-up ramps: A case study

**DOI:** 10.1371/journal.pone.0227943

**Published:** 2020-01-27

**Authors:** Paulo Roberto Laranjeira, Jeane Aparecida Gonzalez Bronzatti, Camila Quartim de Moraes Bruna, Rafael Queiroz de Souza, Kazuko Uchikawa Graziano, Viktoriya Lusignan

**Affiliations:** 1 Cardinal Health, Miami Lakes, FL, United States of America; 2 Nursing School São Paulo, Universidade de São Paulo, São Paulo, SP, Brazil; 3 Research and Development Technician, Getinge, Englewood, CO, United States of America; Public Library of Science, UNITED KINGDOM

## Abstract

**Objective:**

To determine if the standardized B&D type test for hospital steam sterilizer is correctly indicating cycle failures in slower come-up ramps cycles.

**Methods:**

Two commercially available B&D type test were challenged in a quality control sterilizer. A common failure was simulated in triplicate cycles, using a standardized cycle configuration, and then compared to triplicate cycles of a common cycle configuration. Tests procedures were conducted according to B&D manufacturer test standard and results were compared to standardized endpoint specifications.

**Results:**

We found that B&D type tests are only capable of detecting the presence of non-condensable gases if the sterilization equipment is adjusted to meet all the cycle requirements of the test. False positive results were obtained with come-up ramp time of 3 min. Correct results were only obtained with come-up ramp of 1.7–1.9 min.

**Conclusions:**

Until the ISO 17665 and AAMI ST-79 standards are revised, equipment qualification experts should observe come-up time duration criterion for B&D type test cycles according to ISO 11140–4. Sterile Processing Department professionals must add the come-up ramp criterion to cycle evaluation before clearing the equipment for routine use. This will allow B&D correct performance, reducing the infection risk from unsterilized medical device.

## Introduction

Medical devices that are reprocessed in the Sterile Processing Department (SPD) must be disinfected or sterilized, depending on the Spaulding Classification, prior to use in patients, to prevent infection and guarantee patient safety.[1] The preferred and most commonly used sterilization method for medical devices is steam sterilization. This method uses saturated steam as a sterilizing agent to reduce bacterial spore number to a sterile assurance level of 10^−6^.[2,3]

Saturated-steam sterilization cycles employ three monitoring methods to ascertain that all required sterilization conditions are within recommended specifications: physical, chemical, and biological methods. The physical indicators are printed out for each cycle; they are based only on the temperature, pressure, and time data obtained from transducers and sensors installed in the equipment, following standardized specifications.[4] Chemical indicators are produced to react (e.g., color change) to standardized critical process variables (e.g., the time and temperature) at a specific endpoint (e.g., chemical ink on B&D type test for steam sterilizers).[5,6] Biological indicators are considered to provide the real evidence of an effective sterilization cycle because their readouts directly reflect the eradication of a microorganism; their technical characteristics are also standardized.[7]

The presence of air and other non-condensable gases (NCG) inside the chamber during a sterilization cycle is one of the biggest threats to the sterilization process. NCG prevent the steam from reaching the medical device, inhibiting thermal coagulation of microorganisms and spores during the sterilization.[8] Many steam sterilization equipments are not capable of detecting the presence of NCG and the physical indicator (print-out), which only registers pressure, temperature and time, will prompt the SPD professionals to approve a cycle that has finished correctly; however, if NCG were present, the sterilization was compromised.[9]

In 1963, a B&D test was developed to help users determine whether NCG were present during a cycle whose physical parameters were within recommended specifications.[10] The International Organization for Standardization (ISO) created a specific document to standardize the construction and performance of tests for the ready-to-use B&D type tests that are commercially available. [11] Essentially, the original test was composed of a stack of cotton cloths with a cross made of tape (with a type-1 chemical indicator) placed in the middle of the stack.[1] For the B&D test to detect the presence of NCG, an empty chamber cycle, with the exposure phase set to 134°C for 3.5 min, is required.[11] This configuration reflects resistance to steam penetration and air removal. If air is correctly removed and steam is able to penetrate the stack, the chemical indicator on the tape turns black, indicating a correct cycle. The currently commercially available B&D type tests are more compact and ready to use, than the original handmade towel pack, and they are developed and tested to mimic the original B&D test performance.[1]

The B&D test has become a mandatory monitoring tool for the daily release of equipment for use; its usage is referenced in many international technical documents and regulations.[1,12,13] Adequate implementation of the B&D test relies on correct equipment maintenance and the adjustment of cycle parameters.[4,11] In the event of a B&D test failure, the operator should call the maintenance department, but when the technician confirms that the vacuum level and the number of pulses were correctly set, and the equipment has passed qualification according to ISO 17665–1,[14] the technician is not able to determine its cause. However, after altering of the equipment settings (specifically, increasing the come-up ramp duration, i.e., the time between the last vacuum point in the conditioning phase and the beginning of the exposure phase), the B&D type test begins to give a passed result. Herein, we perform a case study to evaluate whether technical interventions may result in false positive B&D approval results, concealing the presence of NCG, and providing incorrect information to SPD and infection prevention professionals. This is because the presence of NCG inside the chamber is not detected by any other chemical process indicator currently used to monitor the sterilization process.

## Materials and methods

This case study was performed at Getinge´s quality control laboratory, located in Englewood, CO, USA. A Getinge 250-L saturated steam sterilizer was used. This equipment had its performance qualified according to the technical specifications for testing the B&D type test, ISO 11140–4.[11] The water used to feed the vacuum pump was maintained at 15°C during all cycles, because higher temperatures affect the attainment of the vacuum level.[4] The water used to produce steam was obtained via reverse osmosis and, before going into the boiler, it was passed through a degasser to remove all NCG. The steam generation system has been challenged using quality test kits for NCG, dryness, and superheat, according to the methodology, apparatus, and criteria specified in the EN 285 document.[4]

### Study design

Basically, three different tests are conducted with a B&D type test, with empty chamber, to verify if the B&D test results satisfy the standard requirements.[11] The first test involves simulating a failure in removing air from the chamber, by lessening the vacuum levels (less efficient air removal). The second test involves inducing an air leak, also causing air removal failure. Since these two situations are easily diagnosed and corrected by a maintenance technician facing a failed B&D test, they were not employed in this study. The one failure condition tested involved a simulation of uncontrolled air inflow into the chamber, resulting in NCG, which is normally overseen by the technician. Since NCG may be present in the steam, either because of residue from reverse osmosis water treatment or a leakage in piping or door gaskets,[8] an air inlet directly in the steam line, close to admission into the chamber, was used, employing the apparatus and criteria specified by ISO 11140–4.[11]

Thermal qualification reports of 95 steam sterilizers, of 29 hospitals in Brazil, were analyzed to determine common come-up ramps, and the minimum observed come-up ramp was of 3 min.

Two brands of B&D type test that met ISO 11140–4 [11] requirements were chosen for this study, TST^™^ single use Bowie-Dick test pack 2352 (Browne, UK) and Getinge Check Bowie-Dick mini pack 134°C 6001155600 (Getinge, Sweden), and the intention is not to compare their performance, but to observe a standardized B&D test performance in a sterilizer. All cycles were run with only one B&D type test in the chamber, positioned on the lower shelf, on top of the drain. Cycle A, the control cycle, was configured according to cycle 1, annex B, in ISO 11140–4 [11]: Air was removed by sub-atmospheric pulsing, with the come-up ramp set to 250 kPa min^-1^, representing a fast come-up time. The configuration of cycle B was the same as that of cycle A, but with 1050 mL of air injected into the steam line during pressurization stage between 75 kPa and 105 kPa, according to item B.1 in ISO 11140–4.[11] The configuration of cycle C was the same as cycle B, but the come-up ramp was set to 80 kPa min^-1^, resulting in a slow come-up time duration of 3 min. The Integrated Come-up Exposure (ICE), whose limits are intended to ensure that steam admission does not contribute to excessive exposure of the indicator to atypical condition,[11] was calculated according to ISO 11140–4 [11] for each cycle. Cycle A was run only once for each brand of B&D type test. Cycles B and C were run three times each for each brand of B&D type test.

## Results

B&D type test results listed on Table 1 demonstrates that the presence of NCG will be concealed if come-up ramp is not set within the range of 100 to 250 kPa per min [11], even if ICE values were below 2312 sK [11]. Adequate B&D type test results were obtained with a come-up time duration ranging from 1.7 to 1.9 min.

**Table 1:** Total B&D type test results, Integrated Come-up Exposure (ICE) value, and come-up ramp duration for each cycle configuration.

Cycle	Browne B&D			Getinge B&D		
	Result	ICE (sK)	Come-up time (min)	Result	ICE (sK)	Come-up time (min)
A	PASS	1842	1.7	PASS	1922	1.8
B	FAIL	1797	1.9	FAIL	1922	1.8
	FAIL	1842	1.7	FAIL	1800	1.9
	FAIL	1839	1.7	FAIL	1922	1.9
C	PASS	1922	3.1	PASS	2312	3.2
	PASS	1942	3.2	PASS	1935	3.1
	PASS	1942	3.3	PASS	2048	3.1

**Fig 1:**
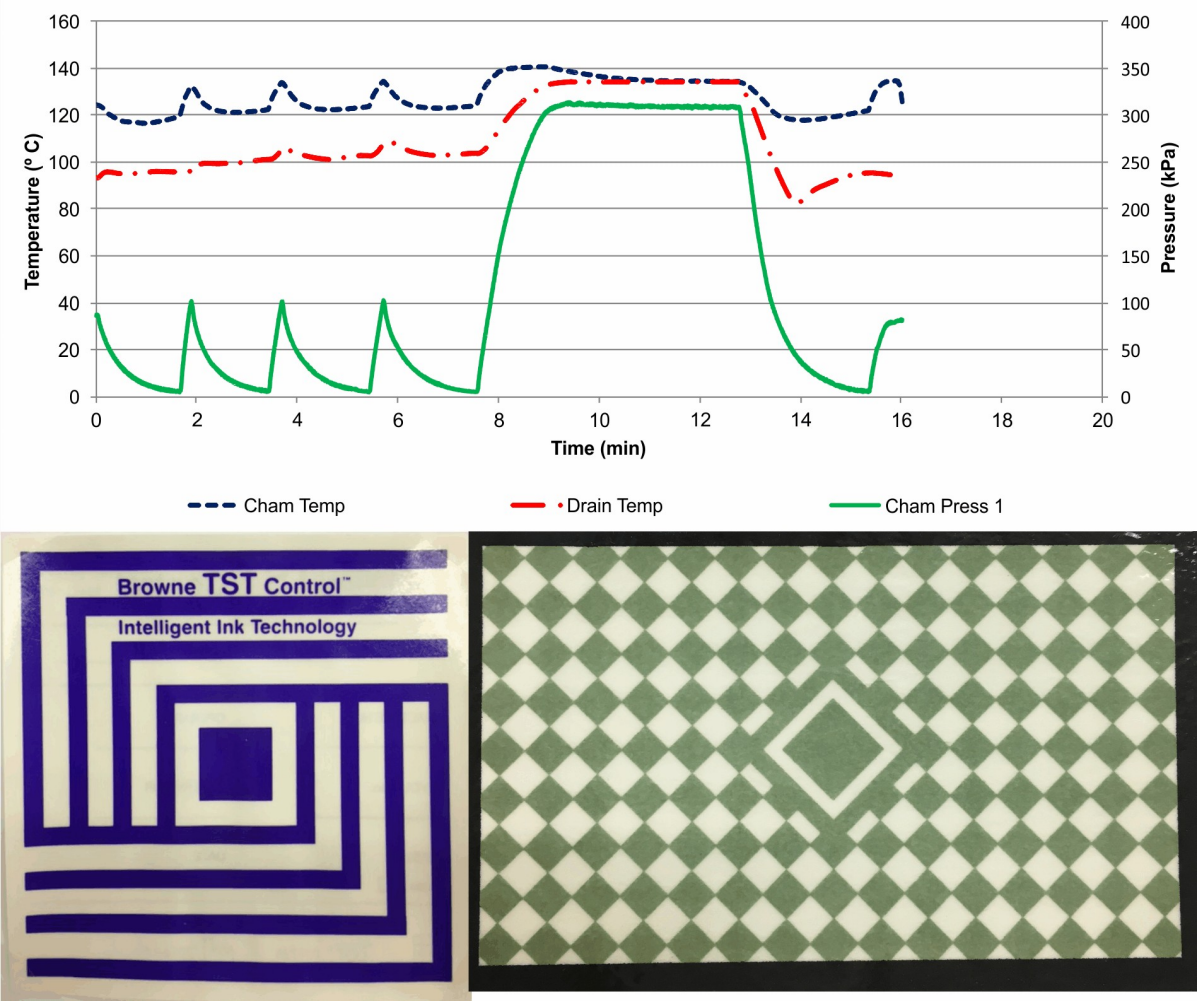
Standard B&D cycle, with sub-atmospheric conditioning phase and fast come-up ramp, and B&D results.

Sterilization cycle graph in Fig 1 (a standard B&D cycle, with sub-atmospheric conditioning phase and fast come-up ramp) shows the temperature measured at the drain and inside the chamber, and pressure during the sterilization cycle. This information is normally available to SPD professionals by way of the physical indicator, as numerical values. B&D type test results are considered together with the required temperature (134°C) and exposure time (3.5 min) recorded by the physical indicator.[15] If the test results indicate approved values, uniform color change of the chemical indicator inside the B&D type test, and the temperature and time requirements are met, the equipment is then cleared for daily routine usage (Fig 1).

**Fig 2:**
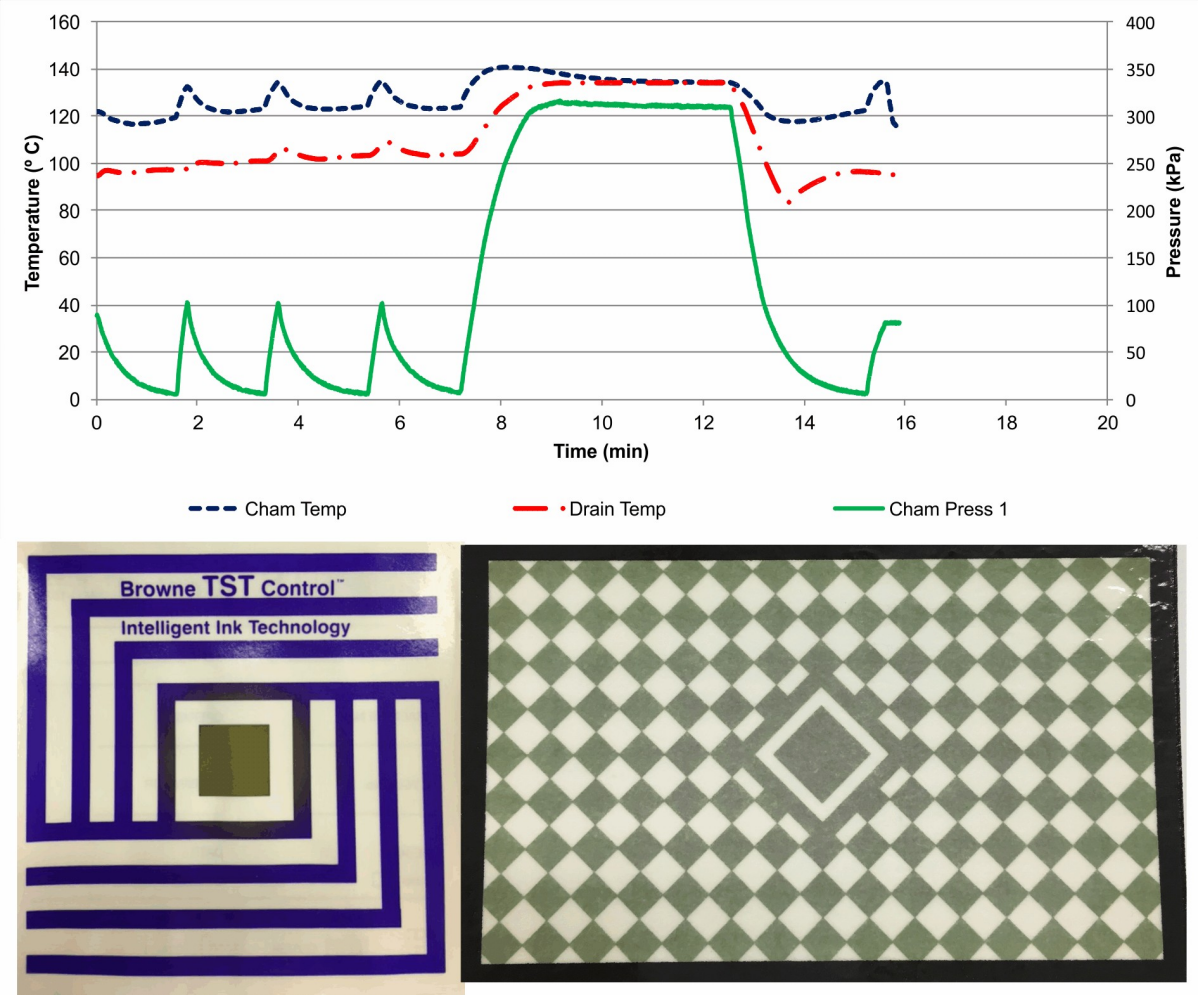
Standard B&D cycle, with sub-atmospheric conditioning phase and fast come-up ramp, with NCG, and B&D results.

As seen in Fig 2 (a cycle with sub-atmospheric conditioning phase and fast come-up ramp, with NCG), the sterilization cycle graph was identical to that in Fig 1. Since this cycle was run with NCG, this indicated that monitoring of only pressure, temperature and time was not capable of detecting the NCG presence, demonstrating the importance of using B&D type tests to verify NCG presence, as demonstrated by the test failure (Fig 2). In this situation, SPD professionals would not release the sterilizer based on B&D type test result, even if physical parameters were within specification.

**Fig 3:**
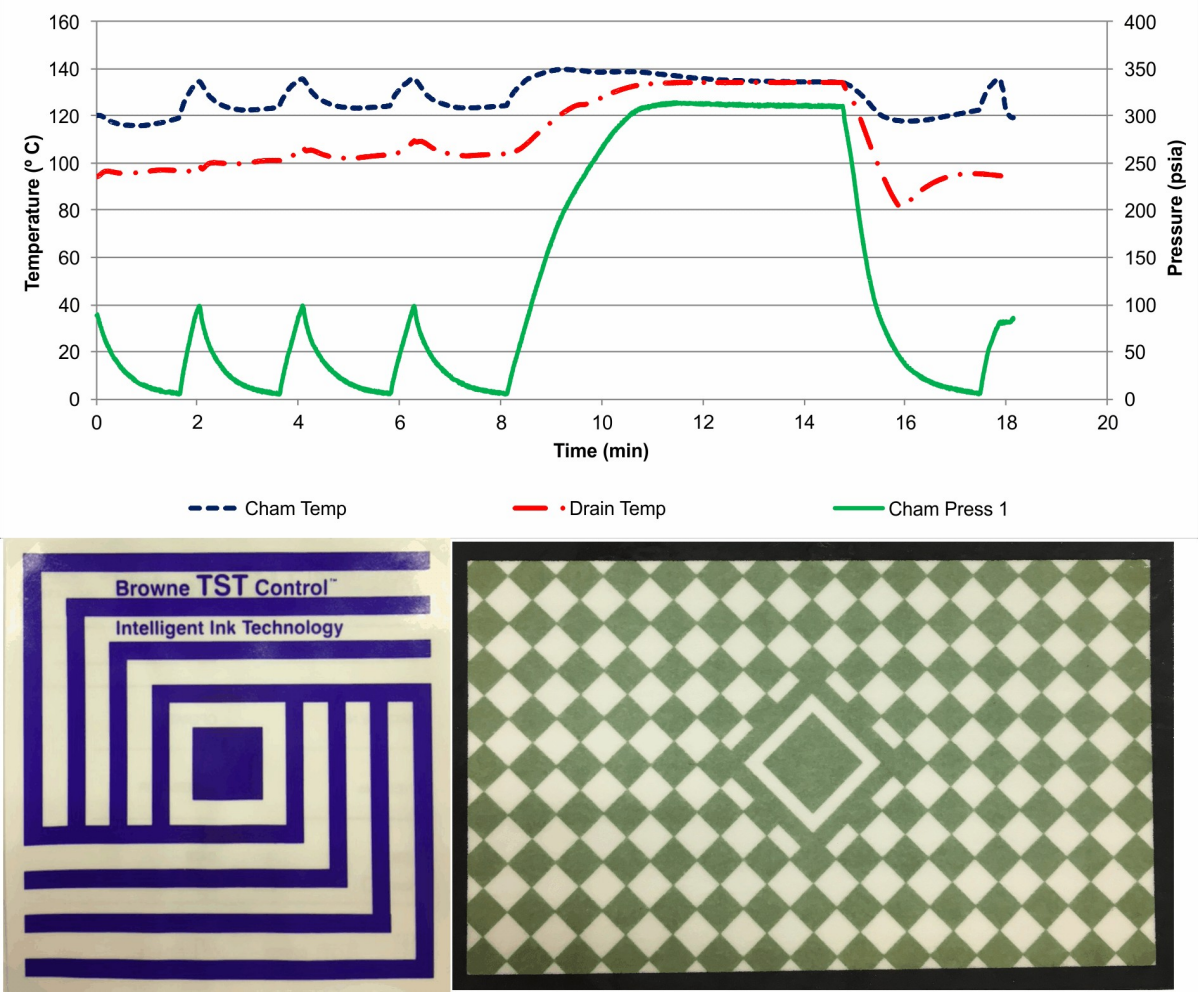
Standard B&D cycle, with sub-atmospheric conditioning phase but slow come-up ramp, and NCG. Representative B&D results for each tested brand are reported below.

By reducing the come-up ramp speed (Fig 3), NCG that were trapped inside the B&D type test had more time to warm-up and, because of the difference in the mass of steam and air, the air moved away from the chemical indicator sheet,[16] allowing the steam to reach it, causing color change, and resulting in a false positive, with NCG present inside the chamber. Another hypothesis for the incorrect color change is that the slower steam diffusion caused by the slower come-up ramp will allow NCG to heat up. The slower come-up ramp is noticeable (Fig 3). SPD professionals would be able to determine the come-up ramp duration time based on the numerical print out of the physical indicator, by simply subtracting the exposure start time from the last conditioning negative pulse. Unfortunately, the come-up time duration is not specified in the equipment qualification standard ISO 17665–2,[17] or in any other technical document. Since the temperature and exposure time were met and the B&D type tests were passed, SPD professionals would clear the equipment for routine use, with NCG in the cycle.

Further, equipment performance printout (physical indicator) is commonly programmed to give the time, temperature, and pressure for a short interval (usually 1 min) during the exposure phase only; the other phases of the sterilization cycle are limited to set points events, making it difficult to observe the duration of the come-up ramp.

## Discussion

Currently, two distinct approaches exist for the qualification of steam sterilization equipment, either a routine performance challenge or after major repair. One relies on the commercially available B&D type tests, process challenge device, and chemical indicators, establishing the equipment approval criteria solely based on the results of these indicators.[1,18] The other follows a technical standard, which also recommends the use of the same commercially available monitoring devices, and also establishes the thermometric, pressure, and time criteria for the exposure phase to determine if the equipment is operating within the allowed parameter range.[17,19] Neither approach considers the impact of the come-up ramp on the B&D type test results to assess that the equipment operates within specifications, even if slower come-up ramps are present (Fig 3). The current study was conducted based on the performance of only one type of the required B&D type test, and has shown that a revision of equipment qualification requirements may be needed. A number of B&D type tests are available in the market which use a combination of barriers for air removal and steam penetration with electronic temperature and pressure monitor, and data processing capability. These systems, when programmed correctly, can analyze more variables during the B&D cycle and will be able to detect cycle configuration failures. Unfortunately, their performance have not yet being standardized and should be included in the revision of the ISO 11140–4.

In conclusion, B&D type test results depend on the adequate duration of the equipment come-up ramp. Longer come-up ramps can mask B&D test failure, creating challenges for the root cause analysis for infection control. Until further revisions of the equipment qualification requirements are made, users are advised to monitor the come-up ramp duration on the sterilizer printout by subtracting the exposure phase start time from the time that the last vacuum point was registered. As shown in the current study, the come-up ramp duration should be 1.7–1.9 min, in empty-chamber cycles, by adjusting the pressure change during this phase to 250 kPa per min. This adjustment is only required for the B&D cycle configuration, because it is run with only the test pack inside the chamber. For regular sterilization loads the come-up ramp will vary according its mass and condensation formation.

## Supporting information

S1 Data (XLSX)

S1 File (DOCX)
