# Supplementary material for: False positive results of Bowie and Dick type test used for hospital steam sterilizer with slower come-up ramps: A case study
Source: PLoS One. 2020 Jan 27;15(1):e0227943. doi: 10.1371/journal.pone.0227943 (PMC6984693; doi:10.1371/journal.pone.0227943)

**Supporting Information**

Cycle A

Browne B&D – Correct pass result


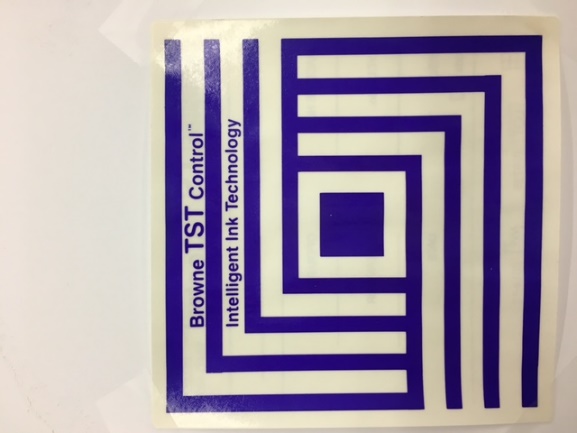


Getinge B&D – Correct pass result


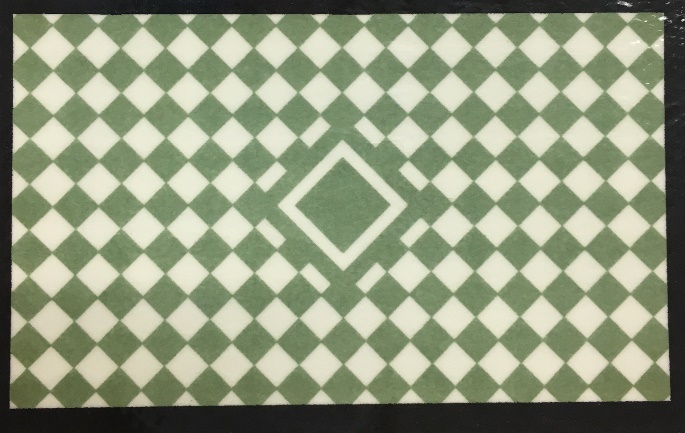


Cycle B1

Browne B&D – Correct failed result


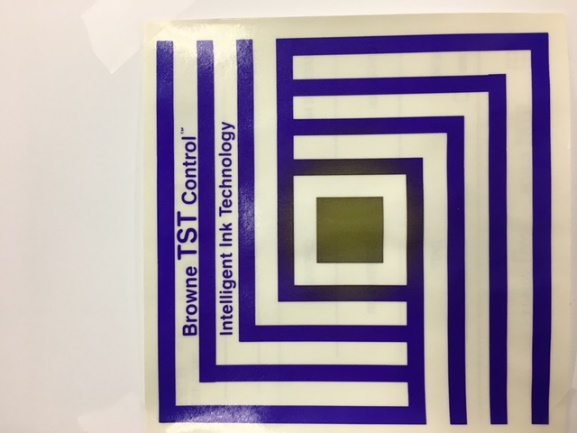


Getinge B&D – Correct failed result


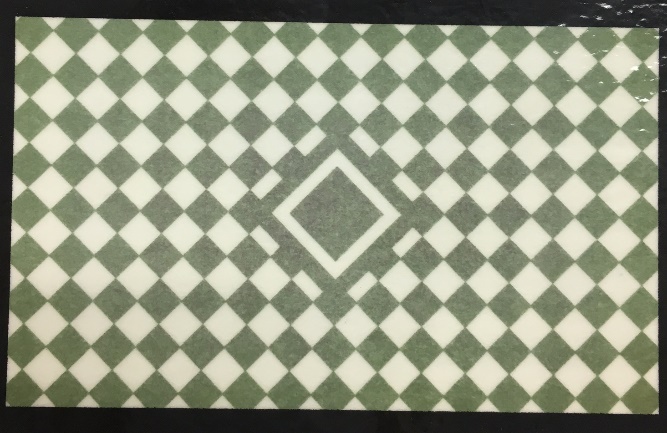


Cycle B2

Browne B&D – Correct failed result


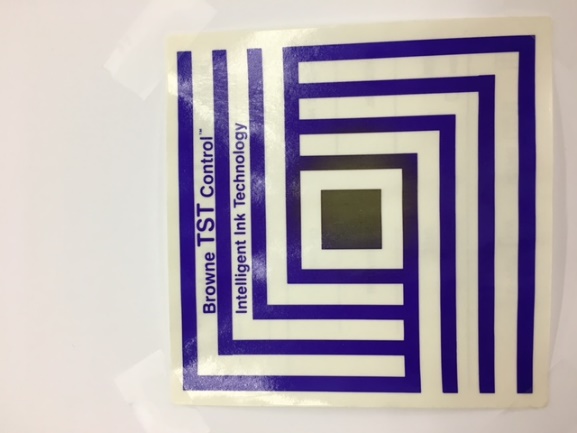


Getinge B&D – Correct failed result


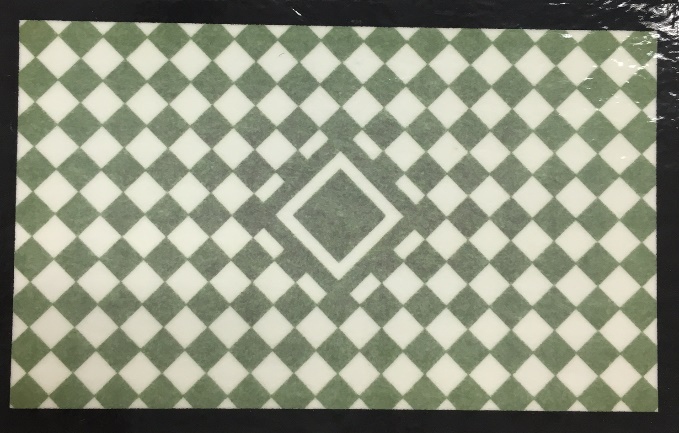


Cycle B3

Browne B&D – Correct failed result


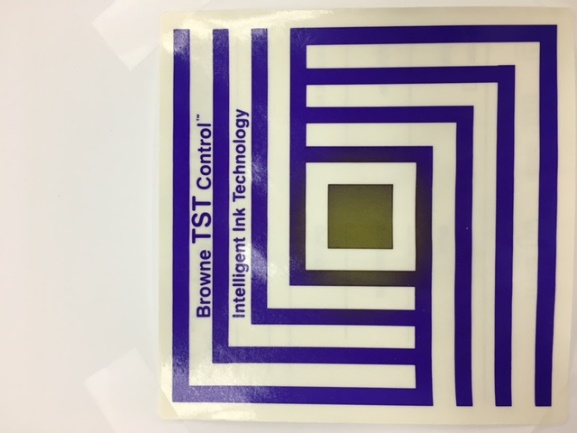


Getinge B&D – Correct failed result


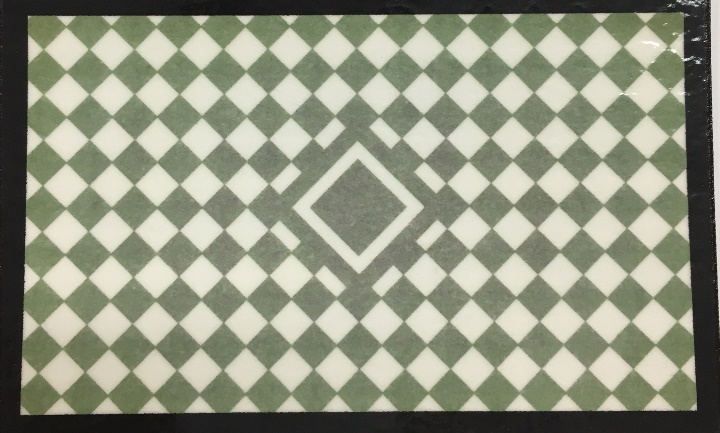


Cycle C1

Browne B&D – Incorrect pass result


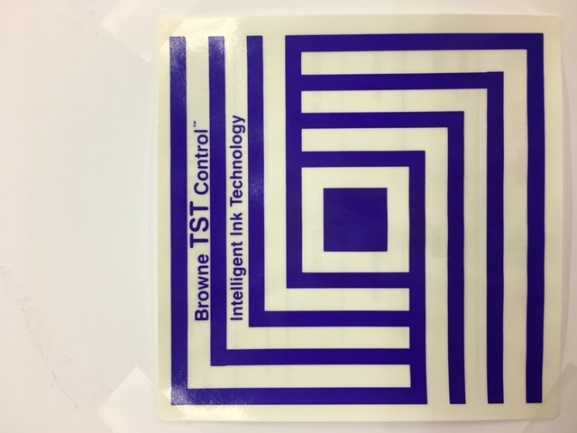


Getinge B&D – Incorrect pass result


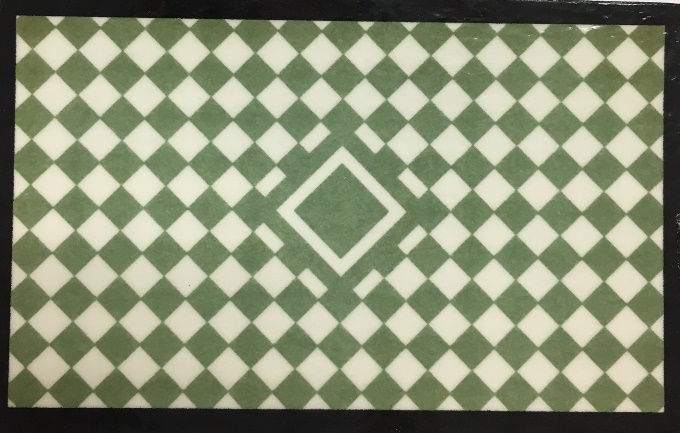


Cycle C2

Browne B&D – Incorrect pass result


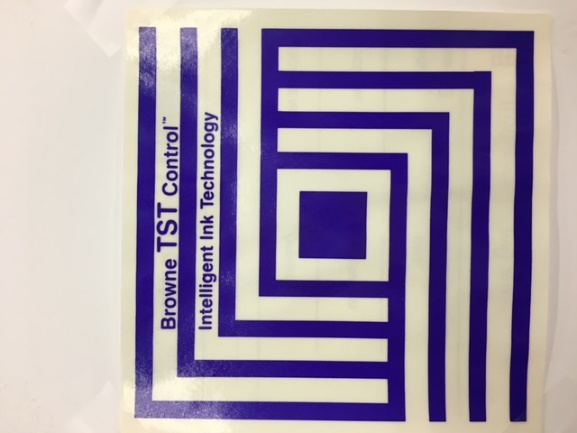


Getinge B&D – Incorrect pass result


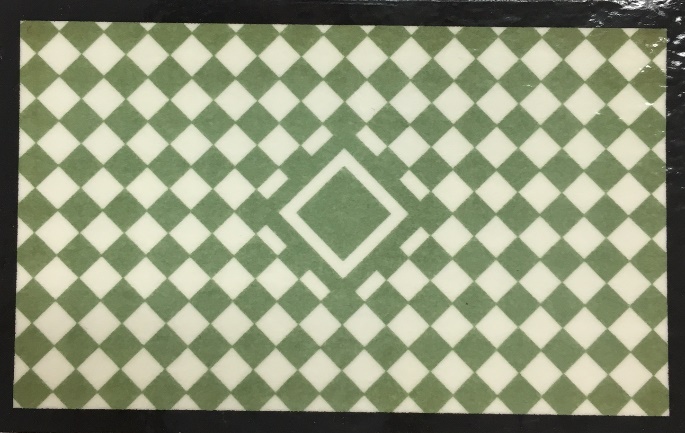


Cycle C3

Browne B&D – Incorrect pass result


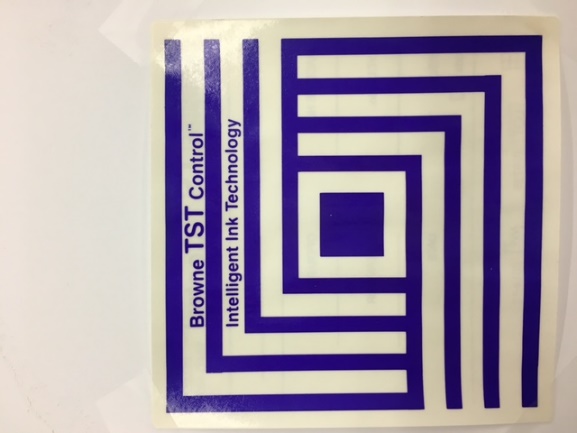


Getinge B&D – Incorrect pass result


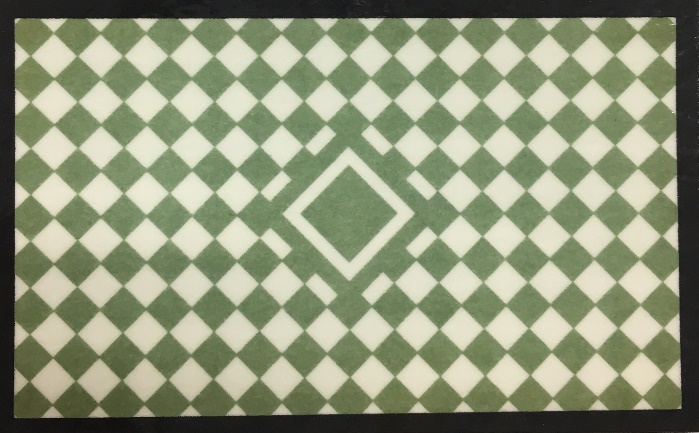

Supplement: S1 File — (DOCX) [file pone.0227943.s002.docx]
